# MiR‐335 overexpression impairs insulin secretion through defective priming of insulin vesicles

**DOI:** 10.14814/phy2.13493

**Published:** 2017-11-09

**Authors:** Vishal A. Salunkhe, Jones K. Ofori, Nikhil R. Gandasi, Sofia A. Salö, Sofia Hansson, Markus E. Andersson, Anna Wendt, Sebastian Barg, Jonathan L. S. Esguerra, Lena Eliasson

**Affiliations:** ^1^ Department of Clinical Sciences Malmö Islet Cell Exocytosis Lund University Diabetes Centre Lund University Malmö Sweden; ^2^ Department of Medical Cell Biology Uppsala University Uppsala Sweden; ^3^ Present address: Department of Molecular and Cellular Endocrinology Beckman Research Institute of City of Hope Duarte California

**Keywords:** Beta cell, exocytosis, insulin secretion, microRNA, patch‐clamp, SNAP25, STXBP1, TIRF, Type 2 Diabetes

## Abstract

MicroRNAs contribute to the maintenance of optimal cellular functions by fine‐tuning protein expression levels. In the pancreatic *β*‐cells, imbalances in the exocytotic machinery components lead to impaired insulin secretion and type 2 diabetes (T2D). We hypothesize that dysregulated miRNA expression exacerbates *β*‐cell dysfunction, and have earlier shown that islets from the diabetic GK‐rat model have increased expression of miRNAs, including miR‐335‐5p (miR‐335). Here, we aim to determine the specific role of miR‐335 during development of T2D, and the influence of this miRNA on glucose‐stimulated insulin secretion and Ca^2+^‐dependent exocytosis. We found that the expression of miR‐335 negatively correlated with secretion index in human islets of individuals with prediabetes. Overexpression of miR‐335 in human EndoC‐*β*H1 and in rat INS‐1 832/13 cells (OE335) resulted in decreased glucose‐stimulated insulin secretion, and OE335 cells showed concomitant reduction in three exocytotic proteins: SNAP25, Syntaxin‐binding protein 1 (STXBP1), and synaptotagmin 11 (SYT11). Single‐cell capacitance measurements, complemented with TIRF microscopy of the granule marker NPY‐mEGFP demonstrated a significant reduction in exocytosis in OE335 cells. The reduction was not associated with defective docking or decreased Ca^2+^ current. More likely, it is a direct consequence of impaired priming of already docked granules. Earlier reports have proposed reduced granular priming as the cause of reduced first‐phase insulin secretion during prediabetes. Here, we show a specific role of miR‐335 in regulating insulin secretion during this transition period. Moreover, we can conclude that miR‐335 has the capacity to modulate insulin secretion and Ca^2+^‐dependent exocytosis through effects on granular priming.

## Introduction

Hyperglycemia and the development of type 2 diabetes (T2D) depend on environmental components together with genetic factors resulting in insulin resistance in the target tissues and reduced capacity of the pancreatic *β*‐cells to secrete enough insulin. The release of insulin is biphasic and evidence have put forward that impaired insulin secretion precedes insulin resistance (Gerich 2002) and that first‐phase insulin secretion is lost already when the patient has impaired glucose tolerance (IGT) or prediabetes (del Prato and Tiengo 2001). In the *β*‐cells, exocytosis serves to release insulin from large dense‐core vesicles in response to elevated cytosolic [Ca^2+^]_i_ (Ammala et al. 1993, Eliasson et al., 2008; Wang and Thurmond 2009). Prior to fusion with the plasma membrane, the granules need to dock to the release site and undergo the process of granular priming to make them ready for release of their insulin cargo. Impaired priming has been suggested to underlie the absence of first‐phase insulin secretion in individuals with pre‐ and full‐blown diabetes (Eliasson et al. 2008).

As in neuronal cells, the assembly of the SNARE (Soluble NSF Attachment protein Receptor)‐proteins, including VAMP2 (vesicle‐associated‐membrane‐protein2), STX1A (Syntaxin1A), and SNAP25 (Synaptosomal‐Associated‐Protein‐of‐25‐kDa), is a key process in insulin exocytosis (Eliasson et al., 2008, Wang and Thurmond 2009; Pang and Sudhof 2010). In addition, several other proteins are required for the specific targeting of secretory granules to the release sites and the regulation of exocytosis (Wang and Thurmond 2009; Pang and Sudhof 2010). These include the Sec1/Munc18 (or syntaxin‐binding protein 1 (STXBP1)) proteins, thought to guide SNARE complex assembly (Gulyas‐Kovacs et al. 2007; Tomas et al. 2008; Rizo and Sudhof 2012), and the synaptotagmins (SYTs) the primary Ca^2+^ sensors of exocytosis (Gauthier and Wollheim 2008; Pang and Sudhof 2010). The expression of many exocytotic genes is down‐regulated in islets from donors with T2D (Ostenson et al. 2006; Andersson et al. 2012) as well as in the spontaneous diabetes model the Goto‐Kakizaki (GK) rat (Gaisano et al. 2002; Zhang et al. 2002).

MiRNAs are small (~20‐nt), nonprotein coding, endogenously expressed RNAs that regulate gene expression (Bartel 2004). MiRNAs guide the RNA‐induced silencing complex (RISC) to target the mRNAs by direct base‐pairing, which may include wobble base‐pairing in mammalian systems, between the miRNA 5′ seed region (~7‐nt long) and their target mRNA sequence usually in the 3′UTR (Brennecke et al. 2005). The regulation of protein output by miRNAs has been suggested to function either as a classical binary off‐switch or as a rheostat, in which miRNAs fine‐tune optimal protein output (Bartel 2009).

In the context of T2D several miRNAs have been shown to directly or indirectly regulate crucial components of glucose‐stimulated insulin secretion and exocytosis in *β*‐cells (Lovis et al. 2008; Eliasson and Esguerra 2014). The exocytotic process involves a plethora of proteins (Eliasson et al., 2008, Wang and Thurmond 2009), several of which are regulated by miRNAs (Lovis et al. 2008; Eliasson and Esguerra 2014; Salunkhe et al. 2015). We have earlier demonstrated up‐regulation of at least 24 miRNAs (Esguerra et al. 2011) in the islets of the GK‐rat, whose hallmark phenotype is impaired glucose‐stimulated insulin secretion (Portha et al. 2009). We found enrichment of transport and secretory genes among the putative targets of the most upregulated miRNAs in GK rat islets. Specifically, we demonstrated that miR‐335 downregulates *Stxbp1* mRNA (syntaxin‐binding protein 1 or Munc18‐1) by direct interaction (Esguerra et al. 2011).

Here we investigated if modulation of miR‐335 expression correlates to altered insulin secretion output in human islets. In addition, we performed a detailed analysis on how miR‐335 overexpression influences the exocytotic process using capacitance measurements, live‐TIRF imaging, and western blot analyses of potential targets. The data highlight the important regulatory role of miRNAs during T2D development.

## Methods

### Ethical approval

The use of human islets from deceased donors was approved by the ethics committees in Malmö and Uppsala, Sweden.

### Cell culture

Rat insulinoma INS‐1 832/13 cells (Hohmeier et al. 2000) was maintained in RPMI 1640 medium containing 11.1 mmol/L glucose (HyClone, UT, USA) as previously described (Salunkhe et al. 2015). EndoC‐*β*H1 cells (EndoCells, Paris, France) (Ravassard et al. 2011; Andersson et al. 2015) were maintained in a culture medium containing: DMEM (5.6 mmol/L glucose), 2% BSA fraction V (Roche Diagnostics, Mannheim, Germany), 10 mmol/L nicotinamide (Merck Millipore, Darmstadt, Germany), 50 *μ*M 2‐mercaptoethanol, 5.5 *μ*g/mL transferrin, 6.7 ng/mL sodium selenite (Sigma‐Aldrich), 100 U/mL penicillin, and 100 *μ*g/mL streptomycin (PAA Laboratories, Pasching, Austria). Cells were tested for mycoplasma regularly.

### Human islets

Islets from 28 human donors (F/M 16/12, age 58.2 ± 1.63, BMI 26.8 ± 0.6 kg/m^2^, HbA1c 5.82 ± 0.11, days in culture 2.8 ± 0.29) were provided by the Nordic Network for Clinical Islet Transplantation (Uppsala, Sweden). Human islets were hand‐picked under microscope to ensure high purity.

### Transfection

One day prior to transfection cells were seeded in antibiotic‐free RPMI 1640 media in a 24‐well‐plate. For transient overexpression, a final concentration of 25 nmol/L chemically modified double‐stranded mature miRNA miR‐335 Pre‐miR™ miRNA Precursor (PM10063, Life Technologies, CA) or Pre‐miR™ miRNA Precursor Negative Control #1 (AM17110, Life Technologies) were used. For down‐regulation, a final concentration of 50 nmol/L of the following was used: miRcury anti‐miR LNA335 (#410201‐00, Exiqon, Denmark), LNA Negative Control B (#199005‐00, Exiqon, Denmark) *Silencer* ^*®*^ *Select Pre‐Designed siRNA* against *Syt11* (s133472, Life Technologies), and *Silencer* ^®^ Select Negative Control No. 2 siRNA (Life Technologies) was used. Transfection was performed according to the manufacturer's protocol using lipofectamine^®^ RNAiMAX Reagent (Invitrogen, CA). Cells were transfected 72 h prior to experiments.

### Glucose‐stimulated insulin secretion

For glucose‐stimulated insulin secretion assay, cells were plated in triplicate for each condition and the assay was performed as described (Salunkhe et al. 2015). For K^+^‐induced insulin secretion the secretion buffer contained 50 mmol/L KCl (adjusted by reducing equimolar amount of NaCl). Insulin secretion measurements were normalized to total protein/well. Protein extraction was performed using RIPA buffer as previously described (Salunkhe et al. 2015). Protein content in cell homogenates was analyzed using BCA assay (Pierce^®^BCA Protein Assay Kit #23227, IL). Secreted and total insulin were measured using Coat‐a‐Count RIA (Millipore Corporation, MA) and Mercodia insulin ELISA (Mercodia, Uppsala, Sweden).

### RNA extraction, RT‐PCR, and qPCR

RNA extraction, RT‐PCR for total RNA, and stem‐loop RT‐qPCR for microRNA was performed as previously described (Esguerra et al. 2011; Salunkhe et al. 2015) using pooled stem‐loop primers from TaqMan^®^MiRNA Assays: hsa‐miR‐335‐5p (#RT_000546), U6 (#RT_001973), and U87 (#RT_001712). The human miR‐335 (miRBase ID: hsa‐miR‐335‐5p/Acc. no. MIMAT0000765) and rat miR‐335 (rno‐miR‐335/Acc.no. MIMAT0000575) have identical mature sequences and were detected by the same Taqman miRNA assay. qPCR for protein coding genes was performed using primers from TaqMan^®^Gene Expression Assays; *Syt11* (Rn00581475_m1) and endogenous controls *Hprt1* (Rn01527840_m1) and *Ppia* (Rn00690933_m1). Relative expressions were calculated using the ΔΔC_t_ method.

### Western blot analysis

Protein extraction and measurement of protein content was performed ~72 h after transfection as described above. Protein samples were separated on 4–15% precast gradient polyacrylamide gels (Bio‐Rad Laboratories, CA) and then transferred to PVDF membranes. The membranes were blocked (at 4°C) with 5% milk and 1% BSA in a buffer consisting of 20 mmol/L Tris, 150 mmol/L NaCl and 0.1% (v/v) Tween‐20 (pH 7.5) for 1 h. Proteins were probed with antibodies for SNAP25 (1:500; #111011, Synaptic Systems, Germany), STXBP1 (1:500; #116002, Synaptic Systems, Germany), SYT11 (1:500; #WH0023208M3 Sigma‐Aldrich, Germany), Beta‐actin (1:1000; #A5441, Sigma‐Aldrich, Germany), and Cyclophilin B (1:2000; #ab16045 Abcam, UK), and incubated overnight at 4°C. The primary antibodies were detected using HRP‐conjugated goat anti‐rabbit/anti‐mouse secondary antibody (1:10,000; #7074S, Cell Signaling Technology) and anti‐mouse immunoglobulins/HRP antibody (1:1000; #P0448, Dako, Denmark). Bands were visualized using SuperSignal West Femto Maximum Sensitivity Substrate (#34096; Thermo Scientific, MA) and AlphaImager (ProteinSimple, CA). Quantification was made using FluorChem SP software (ProteinSimple).

### Electrophysiology

To measure ion channel currents and exocytosis (as changes in membrane capacitance) whole‐cell patch clamp experiments on single cells were performed as previously described (Salunkhe et al. 2015), and with a pipette solution containing (mmol/L): 125 Cs‐Glutamate, 10 NaCl, 10 CsCl, 1 MgCl_2_, 0.05 EGTA, 3 Mg‐ATP, 5 HEPES, and 0.1 cAMP (pH 7.15 using CsOH) and an extracellular solution with (mmol/L): 118 NaCl, 20 TEA‐Cl, 5.6 KCl, 2.6 CaCl_2_, 1.2 MgCl_2_, 5 glucose, and 5 HEPES (pH 7.4 using NaOH). The recordings were performed using patch master software (version 2–73) and EPC‐10 amplifier (Heka Elektronik, Lambrecht, Germany). Exocytosis was measured as changes in cell membrane capacitance, and it was evoked by a train of ten 500‐msec depolarizations from −70 mV to 0 mV applied at 1 Hz. Voltage‐dependent currents were investigated using an IV‐protocol, in which the membrane was depolarized from −70 mV to voltages between −40 mV and +40 mV during 50 msec. All experiments were carried out with constant buffer perfusion at 32°C. The measured voltage‐dependent current consists of Na^+^‐ and Ca^2+^‐current components. The rapid peak‐current (Ip) represents the Na^+^ current and the sustained current (I_sus_), measured during the latter 20 msec of the depolarizations, reflects the Ca^2+^‐current. Charge (Q) was measured ~ 2 msec after the onset of the pulse to exclude the Na^+^‐current and is therefore representative of the Ca^2+^‐influx.

### TIRF microscopy

INS‐1 832/13 cells were plated on coverslips coated with poly‐D‐lysine and immediately cotransfected with mature miR‐335 and the granule marker NPY‐EGFP. Cells were imaged 36 h after plating in a solution containing (in mmol/L) 138 NaCl, 5.6 KCl, 1.2 MgCl_2_, 2.6 CaCl_2_, 10 d‐glucose, 5 Hepes HEPES (pH 7.4 with NaOH), supplemented with 200 *μ*mol/L Diazoxide and 2 *μ*mol/L forskolin. Exocytosis was evoked by timed local application of high K^+^ (75 mmol/L KCl equimolarly replacing NaCl) for 1 min through a pressurized glass electrode, similar to those used for patch clamp experiments. All experiments were carried out with constant buffer perfusion at 32°C.

Cells were imaged using a custom‐built lens‐type total internal reflection (TIRF) microscope based on an Axiovert 135 microscope with a 100x/1.45 objective (Carl Zeiss). Excitation was from a DPSS laser at 491 (Cobolt, Stockholm, Sweden), controlled with an acousto‐optical tunable filter (AA‐Opto, France) and using dichroic Di01‐R488/561 (Semrock) and emission filter FF01‐523/610 (Semrock). Scaling was 160 nm per pixel and exposure time 100 msec per frame at 10 frames/sec.

### Data analysis

In the TIRF microscopy imaging experiments exocytosis events were found by eye. The moment of exocytosis was defined as the first significant change (2 SD) from the preexocytosis baseline. This definition applied both types of event, with or without preceding flash. The decay time was then defined as the time from exocytosis until the signal reached less than one‐third of the amplitude of the event. Traces were read out as ΔF, defined as average fluorescence in a 0.5 *μ*m circle minus the average fluorescence in a surrounding annulus of 0.8 *μ*m. The point of exocytosis was calculated by fitting the granule fluorescence during exocytosis with a discontinuous function, which assumes constant fluorescence before fusion, an inverted exponential decay just after fusion, and finally exponential decay during content release.

(1) $$c=A_{1}$$

for *t < t*_*1*_

$$c=A_{2}+\left(A_{1}-A_{2}\right)e^{\frac{x-x_{2}}{t_{1}}}$$

for *t*_2 _> *t *≥ *t*_1_

$$c=A_{3}+\left(\left(A_{2}+\left(A_{1}-A_{2}\right)e^{\frac{x_{2}}{t_{1}}}\right)-A_{3}\right)e^{\frac{x-x_{2}}{t_{2}}}$$

for *t* ≥ *t*_2_,where *t* is time; c is average fluorescence in a 0.48‐*μ*m‐wide circle at the granule site; *A*_*1*_, *A*_*2*_, and *A*_*3*_ are the fluorescence values at the plateaus; *τ*_1_ and *τ*_2_ are the decay constants for the fluorescence increase after fusion and content release; and *t*_1_ and *t*_2_ are the times of fusion and release, respectively.

### Statistics

Data are presented as mean ± SEM and statistical differences between groups were tested using two‐tailed Student's *t*‐test unless specified otherwise.

For the human islets, a simple linear regression analysis was performed on the miR‐335 expression and stimulatory index data (a measure of insulin secretion capacity in the human islets). F‐test was used to determine significance at *P* < 0.05 as implemented in IBM SPSS v.22.

## Results

### Correlation of miR‐335 expression and insulin secretion in human islets

**Figure 1:**
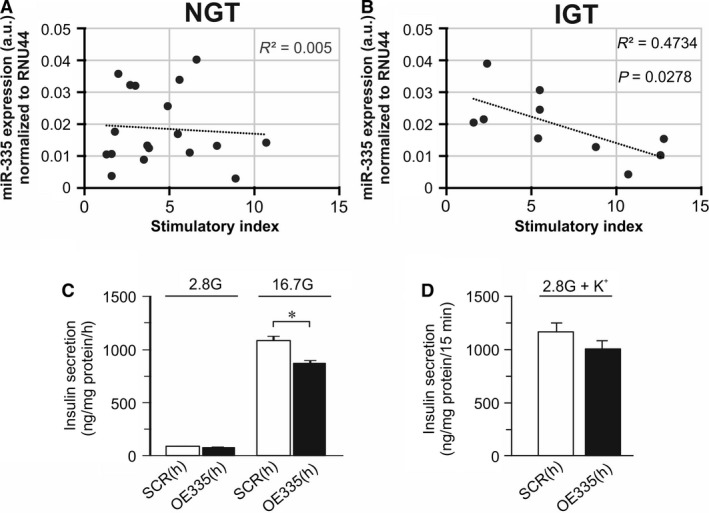
miR‐335 and insulin secretion in human islets and EndoC‐*β*H1 cells. (A) miR‐335 expression in human islets NGT donors against stimulation index, (B) As in A, but data are measured in human islets from IGT donors. (C) Insulin secretion in EndoC‐*β*H1 cells overexpressing miR‐335 (OE335(h); black bars) and in control cells (SCR(h); white bars) cells after stimulation for 1 h in 2.8 mmol/L or 16.7 mmol/L glucose (2.8G and 16.7 G) as indicated. *n* = 3; *P < 0.05. (D) As in C, but insulin secretion was measured after 15 min stimulation in 2.8 mmol/L glucose (2.8G) in the absence and presence of 50 mmol/L KCl (K^+^).

We first investigated miR‐335 expression in human islets. For this we used miR‐335 expression data from 18 donors with HbA1c within the normal glucose tolerance levels (NGT; HbA1c = 5.50 ± 0.06), and 10 donors with high HbA1c corresponding to impaired glucose tolerance levels (IGT; HbA1c = 6.41 ± 0.15, *P* = 7.69E‐06 vs. NGT). MiR‐335 expression negatively correlated with insulin secretion, in islets from donors with IGT, whereas there was no correlation in islets from NGT donors (Fig. 1A–B). In line with these data, the human *β*‐cells EndoC‐*β*H1 overexpressing miR‐335 had reduced glucose‐stimulated insulin secretion (Fig. 1C). There was also a tendency of reduced K^+^‐induced secretion in these cells although this did not reach significance (Fig 1D).

### Decreased expression of STXBP1, SNAP25, and SYT11 after overexpression of miR‐335

**Figure 2:**
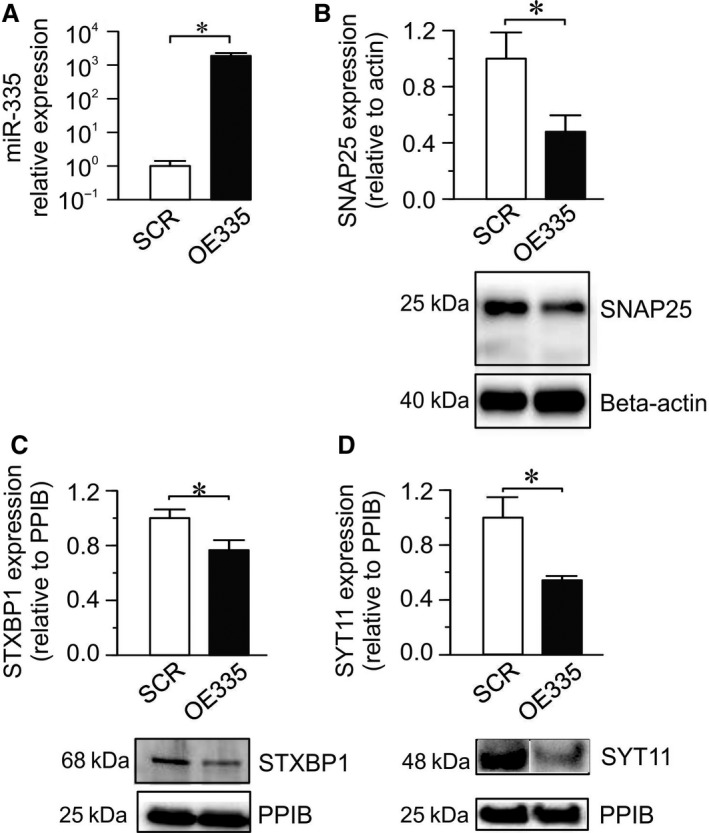
Overexpression of miR‐335 and its effect on the expression of three of its putative protein targets. (A) Expression of miR‐335 using mature miR‐335 mimic (OE335; black bar) relative to its expression using scramble control (SCR; white bar). Mir‐335 expression was normalized to endogenous controls U6 and U87. (*n* = 4; *P < 0.05). (B, C, D). Average protein levels of SNAP25, STXBP1 and SYT11 in OE335 (black bar) compared to SCR (white bar) cells (*n* = 4 each group; *P < 0.05). Representative western blots are shown for each protein. Expression levels were normalized to beta‐actin or cyclophilin B (PPIB).

To investigate the functional role of miR‐335 in *β*‐cells we overexpressed mature miR‐335 in the rat INS‐1 832/13 *β*‐cell line (Fig. 2A). Hereafter these cells are referred to as OE335 cells and the controls are referred to as SCR (scrambled) cells.

Using TargetScan prediction tool (http://www.targetscan.org/) we found several exocytosis genes to be putative targets of miR‐335, both in rodents and/or humans, including the exocytosis genes *Snap25*,* Syt11,* and *Stxbp1*, which we decided to investigate further. Overexpression of miR‐335 significantly reduced protein levels of all these miR‐335 targets, SNAP25, STXBP1, and SYT11 (Fig. 2B–D).

### Overexpression of miR‐335 leads to reduced glucose‐stimulated insulin secretion and exocytosis in INS‐1 832/13 cells

**Figure 3:**
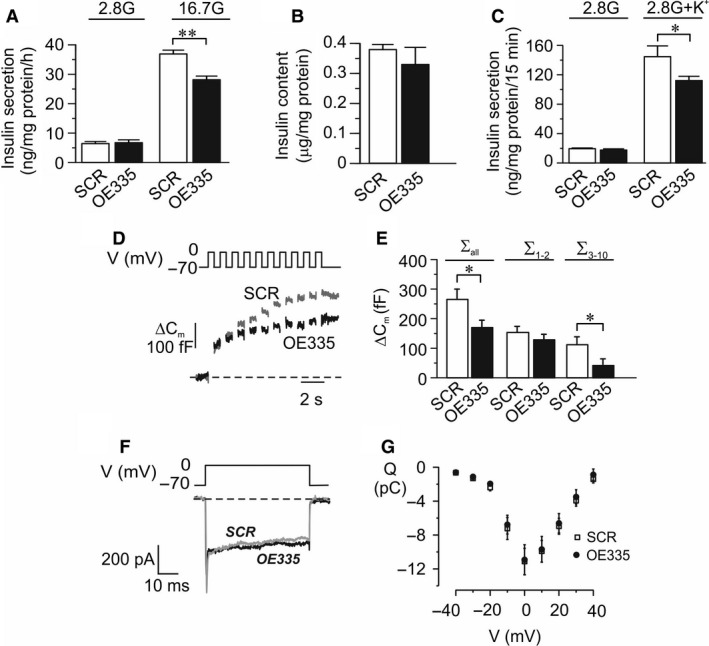
Consequence on insulin secretion and exocytosis in INS‐1 832/13 cells by miR‐335 overexpression. (A) Insulin secretion in OE335 (black bars) and SCR (white bars) cells after stimulation for 1 h in 2.8 mmol/L or 16.7 mmol/L glucose as indicated. *n* = 3; **P < 0.01. Secreted insulin is measured using human insulin‐RIA.(B) Insulin content in OE335 (black bar) and SCR (white bars) cells. *n* = 3. (C) Insulin secretion in OE335 (black bars) and SCR (white bars) cells after stimulation for 15 min. 2.8 mmol/L glucose with or without 50 mmol/L K^+^ as indicated. *n* = 5; *P < 0.05. (D) Representative traces of depolarization‐induced increases of membrane capacitance in OE335 (black trace) and SCR (gray trace) cells. (E) Summary of capacitance changes presented as the summed increased in membrane capacitance during all ten depolarizations (ΣDepol_all_), increase evoked by the first two depolarizations (ΣDepol_1‐2_) or the latter eight depolarizations (ΣDepol_3‐10_). *n* = 13 for OE335 (black bar) and *n* = 16 for SCR (white bar) cells; *P < 0.05. (F) Representative traces of a voltage‐dependent Ca^2+^ currents evoked by a depolarization from −70 mV to 0 mV in a control cell (SCR) and a cell overexpressing miR‐335 (OE335), respectively. (G) Summary of the charge (Q)‐voltage (V) relationship. *n* = 15 for SCR and *n* = 17 for OE335 cells.

As in the human EndoC‐*β*H1 cells, OE335 cells displayed decreased glucose‐stimulated insulin secretion compared to SCR cells (Fig. 3A), without any differences in insulin content (Fig 3B). K^+^‐induced insulin secretion was likewise reduced in OE335 cells (Fig. 3C), indicating effects on exocytosis. We used the patch clamp technique to measure depolarization‐induced exocytosis in OE335 cells (Fig. 3D–E). We found a significantly reduced increase in membrane capacitance elicited by the train of depolarizations (∑Depol_all_) in OE335 cells as compared to SCR (Fig. 3E). The increase in membrane capacitance evoked by the first two depolarizations (∑Depol_1‐2_), was not affected, but the capacitance increase due to the latter depolarizations (∑Depol_3‐10_) was reduced by ~60% in OE335 cells (Fig. 3E). The average cell size did not differ significantly between OE335 and SCR cells (5.91 ± 0.38 pF *vs*. 5.44 ± 0.27 pF; *n* = 13–16). The close link between exocytosis and influx of Ca^2+^ through voltage‐gated Ca^2+^‐channels (Ammala et al. 1993) made us investigate the effect of miR‐335 modulation on the current–voltage relationships of the voltage‐dependent Ca^2+^‐currents in OE335 cells (Fig. 3F–G). We observed no differences in the Ca^2+^‐current at any of the voltages tested.

**Figure 4:**
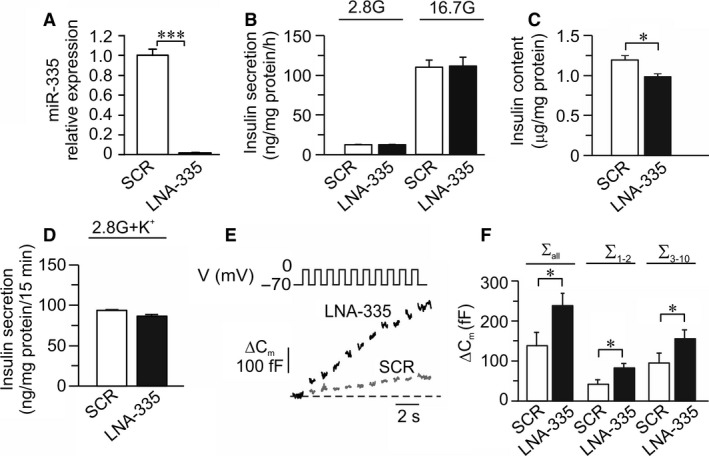
Influence of miR‐335 knockdown on insulin secretion and exocytosis in INS‐1 832/13 cells. (A) Expression of miR‐335 in cells after knockdown of miR‐335 by LNA against miR‐335 (LNA335; black bar) relative to expression in scramble control cells (SCR; white bar). *n* = 4; ***P < 0.001. (B) Insulin secretion in LNA335 (black bars) and SCR (white bars) cells after stimulation for 1 h in 2.8 mmol/L or 16.7 mmol/L glucose as indicated (*n* = 4). The amount of released insulin is measured using human/rat insulin‐ELISA. (C) Insulin content in LNA335 (black bars) and SCR (white bars) cells. *n* = 8; *P < 0.05. (D) Insulin secretion in OE335 (black bars) and SCR (white bars) cells after stimulation for 15 min. in 2.8 mmol/L glucose with 50 mmol/L K^+^. *n* = 4. (E) Representative traces of depolarization‐induced increases of membrane capacitance in LNA335 (black trace) and SCR (gray trace) cells. (F) Summary of capacitance changes presented as the summed increased in membrane capacitance during all ten depolarizations (ΣDepol_all_), increase evoked by the first two depolarizations (ΣDepol_1‐2_) or the latter eight depolarizations (ΣDepol_3‐10_). *n* = 10 for LNA335 (black bar) and *n* = 9 for SCR (white bar) cells; *P < 0.05

We also reduced expression of miR‐335 using locked nucleic acid (LNA) against miR‐335 (LNA‐335; Fig 4A) and investigated the effects on insulin secretion and content (Fig. 4B–D), and on Ca^2+^‐induced exocytosis (Fig. 4E–F). LNA335 reduced insulin content (Fig. 4C), but had no effect on insulin secretion in INS‐1 832/13 cells (Fig 4B and D). However, LNA‐335 significantly increased membrane capacitance elicited by the train of depolarizations (∑Depol_all_). Reduction in miR‐335 expression significantly increased both ∑Depol_1‐2_ and ∑Depol_3‐10_ as compared to control (Fig. 4E–F).

### Overexpression of miR‐335 reduces exocytosis and accelerates content release from individual granules in INS‐1 832/13 cells

**Figure 5:**
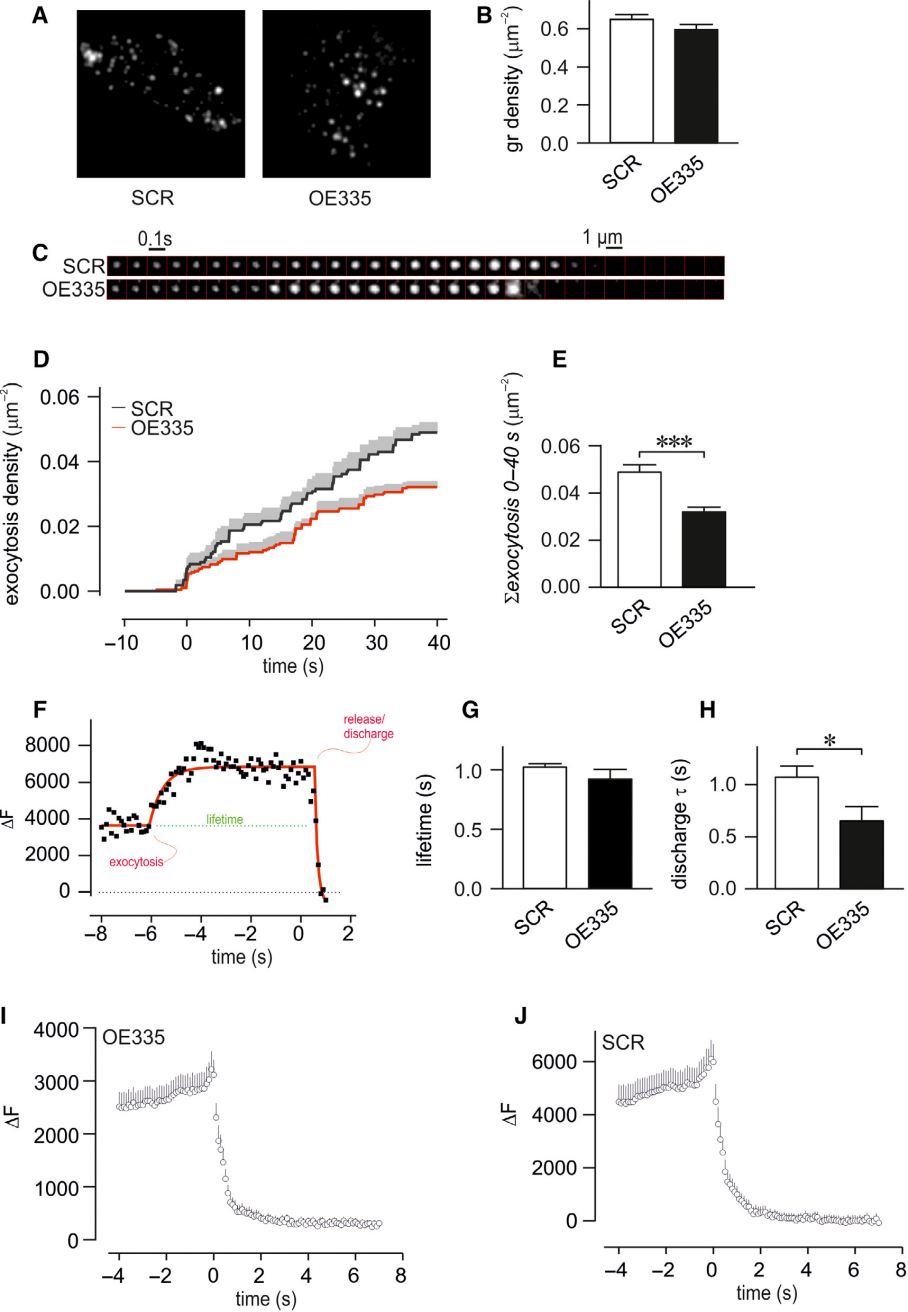
Function of miR‐335 on density of granules and exocytosis measured by TIRF microscopy in INS‐1 832/13 cells. (A) Representative TIRF images of OE335 and SCR cells co‐transfected with NPY‐mEGFP. (B) Density of granules in OE335 (black bar; *n* = 31) and SCR (white bar; *n* = 53) cells. (C) Examples of single exocytosis event measured in OE335 and SCR cells, frames are 100 msec apart. Exocytosis was stimulated through depolarization using elevated K^+^. (D, E) Cumulative number of exocytosis events per area in OE335 and SCR cells (*n* = 8 each group; ***P < 0.001). (F) Representative image of fluorescence time course during a single exocytosis event (black) with fit overlaid (red) illustrating the numerical analysis. The interval between the moments of exocytosis/fusion and release is taken as fusion pore lifetime (green). (G, H) Summary of fitted exocytosis and decay constants of individual events as described in (F) The lifetime of the events (*P=*n*.S.) and decay constant from individual granules during content release (*P < 0.05) was measured in OE335 and SCR cells (*n* = 8 each group). (I,J) Average fluorescence signal of granule fluorescence for exocytosis events in OE335 and SCR cells as in C.

To study in detail the effects of elevated miR‐335 levels on insulin granule docking and release, OE335 and SCR cells were cotransfected with granular marker neuropeptide‐Y (NPY)‐mEGFP, and imaged by TIRF microscopy. Docked granules were present in both groups and their density was similar to previous estimates in these cells (Gandasi and Barg 2014), with 0.59 ± 0.02 *μ*m^‐2^ (*n* = 31) in OE335 and 0.64 ± 0.02 *μ*m^‐2^ (*n* = 53) in SCR (Fig. 5A–B).

We stimulated exocytosis by local application of elevated K^+^, which induces rapid exocytosis of docked and primed granules seen as rapid disappearance of granule fluorescence (Fig. 5C). Two thirds of the exocytotic events were preceded by a temporary increase in granule fluorescence (Fig. 5C, I–J) due to unquenching of EGFP‐fluorescence that occurs when the granule lumen makes aqueous contact with the extracellular medium. Lack of this feature indicates rapid release and implies instantaneous widening of the fusion pore (Barg et al. 2002). The duration of the transient increase, which reflects fusion pore lifetime, was estimated by nonlinear fit of a discontinuous function to the fluorescence time course (Fig. 5F and methods); no significant difference between OE335 cells and SCR cells was detected (Fig. 5G). The kinetics of content release was analyzed by fitting a single exponential function to the fluorescence decay of individual events (Fig. 5F–H). The decay of vesicular fluorescence was twice as fast in OE335 cells compared to SCR cells (Fig. 5H). The total number of exocytosis events in OE335 was significantly less than in SCR cells (Fig. 5D–E).

## Discussion

The contribution of miRNAs in the regulation of pancreatic *β*‐cell function has been a topic of great interest in diabetes research. Consequently, mechanistic details on how dysregulated miRNA expression in the *β*‐cell contributes to the pathophysiology of T2D are beginning to emerge. We have previously shown that miR‐335 is abnormally overexpressed in the pancreatic islets of the GK rat (Esguerra et al. 2011), and we have validated *Stxbp1* mRNA as a direct target of miR‐335. Here we show a negative correlation between miR‐335 expression and insulin secretion in human islets from donors with IGT and provide evidence that overexpression of miR‐335 results in (1) downregulation of three exocytosis protein targets: STXBP1, SNAP25, and SYT11, and (2) impaired exocytosis of insulin granules and decreased insulin secretion.

Although it is known that the defective insulin secretory capacity can be due to defects in the exocytotic machinery, for example, through reduced expression of exocytosis proteins in the GK‐rat (Zhang et al. 2002), it remains unclear how *β*‐cell exocytosis in general is influenced by dysregulated expression of specific miRNAs. Our data support the hypothesis that the main function of miR‐335 is in the regulation of the final stages of insulin secretion. Indeed, both single‐cell capacitance measurements (Fig. 3D–E) and TIRF microscopy data (Fig. 5) confirmed defective priming of already docked granules and deficiencies in postpriming processes of exocytosis after overexpression of miR‐335. The expression of miR‐335 is >1000 times the endogenous levels, prompting us to perform experiments in which the endogenous levels of miR‐335 were silenced (Fig. 4). In these experiments exocytosis was instead increased confirming that miR‐335 is indeed involved in the regulation of *β*‐cell exocytosis. However, while LNA‐335 increased exocytosis, it simultaneously reduced insulin content. The reduced insulin content after miR‐335 knockdown was to some extent surprising and shows that the knock‐down of miR‐335 needs to be adjusted if it should be used therapeutically. The summed outcome of reduced insulin content and increased exocytosis is unchanged insulin secretion in LNA‐335 cells. Our results demonstrate the inherent complexity by which a single miRNA can influence the regulation of multiple targets and hence the overall targeted cellular process. Classically miRNAs has been thought to act as binary off‐switches to repress mRNA expression; models that are more recent suggest that miRNAs can act as rheostats to control protein levels enabling customized expression in different cells and cellular processes (Bartel 2009). Thus, it seems like miR‐335 differentially regulate targets within processes controlling insulin content and insulin exocytosis. Altogether, we can still conclude that the overexpression of miR‐335 reduce insulin secretion mainly through an effect on exocytosis.

We asked if we could identify potential targets of miR‐335 within the exocytosis pathway and if the overexpression reduced the protein level of these targets. Indeed, we found reduced protein expression of SNAP25, STXBP1, and SYT11 in OE335 cells. SYT11 is one of six Ca^2+^‐insensitive isoforms of the synaptotagmin family (Sudhof 2004; Gauthier and Wollheim 2008; Milochau et al. 2014). The cellular function of SYT11 is beginning to be understood (Andersson et al. 2012; Arango Duque et al. 2013; Fadista et al. 2014; Milochau et al. 2014), but details regarding its role in exocytosis remain unclear. Recently SYT11 was also shown to interact with components of the RNA‐induced silencing complex (RISC) and proteins involved in endoplasmic reticulum/Golgi derived‐granule transport in rat beta (INS‐1E) cells implicating SYT11 in both gene regulation by miRNAs and membrane trafficking (Milochau et al. 2014). Although less is known about SYT11 our data presented here are in agreement with the established roles of SNAP25 and STXBP1 as essential components in priming and fusion of insulin‐containing granules (Zhang et al. 2000, 2002; Kang et al. 2002; Ohara‐Imaizumi et al. 2004b, Gulyas‐Kovacs et al. 2007; Eliasson et al., 2008; Tomas et al. 2008). STXBP1 binds to the folded conformation of STX1A during docking (Han et al. 2011), and subsequently dissociates from STX1A upon Ca^2+^ influx to allow formation of the SNARE complex (Tomas et al. 2008) in the priming step (Gulyas‐Kovacs et al. 2007). In addition, studies have implicated STXBP1, as well as SNAP25 and the synaptotagmin isoforms 1 and 7, in the recruitment of granules to the membrane (Gulyas‐Kovacs et al. 2007; Ohara‐Imaizumi et al. 2007; Tomas et al. 2008; de Wit et al. 2009; Dolai et al. 2016). Indeed, granule docking coincides with the formation of clusters containing STXBP1 (Gandasi and Barg 2014) and the physiological importance of docking in insulin secretion was shown in the diabetic GK‐rat (Ohara‐Imaizumi et al. 2004a). Although STXBP1, SNAP25, and SYT11 were down‐regulated in the OE335 cells, we did not observe a reduced number of docked granules. This lack of effects on docking might be due to insufficient miR‐335‐mediated knockdown (Fig. 2B–D) or the possibility that miR‐335 has other unidentified targets that normally inhibit granule docking. It is possible that other targets of miR‐335 contribute to the regulation of insulin secretion, resulting in a net cumulative negative effect of miR‐335 on insulin secretion. For instance, miR‐335 directly targets the transcription factors FOXA2 and SOX17 and miR‐335 thereby promotes mesendodermal lineage segregation (Yang et al. 2014). FOXA2 is not only involved in developmental *β*‐cell differentiation, but the protein also impact *β*‐cell function. Foxa2 deficient mice have increased insulin secretion and increased number of docked granules (Gao et al. 2007). Nevertheless, previous and current findings link miR‐335, SNAP‐25, STXBP1, and SYT11 to the exocytotic process where imbalances in any of them could contribute to impaired insulin exocytosis.

We provide data demonstrating that overexpression of miR‐335 in the human EndoC‐*β*H1 cells reduce insulin secretion and that miR‐335 expression was negatively correlated with insulin secretion in human islets from individuals with prediabetes. It is remarkable that miR‐335 expression levels negatively correlated with insulin secretion only in islets from high HbA1c donors. We can only speculate that as fine‐tuners of cellular processes (Bartel 2009), miRNAs may be mostly required during nonoptimal conditions, perhaps to be able to compensate for the detrimental effects brought about by pathophysiological conditions. In our previous study (Esguerra et al. 2011) we showed elevated miR‐335 expression in GK‐rat as compared to Wistar‐rat islets. This was true also when islets were incubated in hypoglycemic conditions (2.8 mmol/L glucose) for 24 h. However, under hyperglycemic conditions (16.7 mmol/L glucose) for the same period we found evidence of compensatory mechanisms bringing the levels of miR‐335 in the GK‐islets toward that of control. Interestingly, in short‐term incubation for 1 h miR‐335 expression in the GK‐islets was instead elevated after incubation in 16.7 mmol/L glucose as compared to 2.8 mmol/L glucose (or the Wistar control). We postulate therefore that under constant hyperglycemic environment in diabetic patients, miRNA‐mediated regulatory effects are more pronounced and hence the negative correlation between miR‐335 levels and HbA1c is more apparent in the diseased state.

We find our human data of specific interest since one of the signatures of T2D is reduced first‐phase insulin secretion prior to full‐blown diabetes (Gerich 2002). On a cellular level first‐phase insulin secretion has been suggested to be associated with exocytosis and the fusion of primed granules (Rorsman and Renstrom 2003, Eliasson et al., 2008). More so, our observation might be essential in a larger metabolic perspective. Expression of miR‐335 has been demonstrated to be increased in liver and white adipocyte tissue in ob/ob and db/db mice (Nakanishi et al. 2009; Oger et al. 2014), and in islets of diabetic GK‐rats (Esguerra et al. 2011). MiR‐335 is suggested to be involved in lipid metabolism, adipogenesis differentiation, and adipose inflammation (Nakanishi et al. 2009; Zhu et al. 2014), and miR‐335 expression is proposed to be regulated by adipokines (Zhu et al. 2014). It is well‐established that regulation of glucose‐transporters in adipose tissue, involves exocytosis proteins (Cheatham 2000). Hence, our observation that LNA335 and OE335 influence the exocytosis process might not only be valid for pancreatic *β*‐cells, but also for other metabolically relevant tissues. In this aspect it is noteworthy that miRNAs, as important players in gene regulation, have the potential to be innovative therapeutic drug targets against diabetes and associated complications (Eliasson and Esguerra 2014). To achieve this goal, a necessary first step is to understand the mechanistic principles by which miRNAs participate in cellular processes.

In conclusion, our data demonstrate the cellular mechanisms by which miR‐335 influence insulin secretion. Based on our observations we propose that miR‐335 overexpression negatively affects insulin secretion via the reduction in multiple exocytosis protein targets and impaired priming of insulin granules.

## Conflict of Interests

The authors declare that there is no duality of interest associated with this manuscript.
